# Safety of One-Piece Hydrophilic Acrylic Intraocular Lenses in the Ciliary Sulcus

**DOI:** 10.3390/jcm14061972

**Published:** 2025-03-14

**Authors:** Tal Sharon, Naama Lippin, Veronika Yehezkeli, Nimrod Dar, Avner Belkin, Ehud I. Assia

**Affiliations:** 1 Department of Ophthalmology, Meir Medical Center, Kfar Saba 4428164, Israel; 2 Sackler School of Medicine, Tel Aviv University, Tel Aviv 6997801, Israel; 3 Schulich School of Medicine and Dentistry, Western University, London, ON N6A 5C1, Canada; 4 Ein-Tal Eye Center, Tel Aviv 6997801, Israel

**Keywords:** uveitis-glaucoma-hyphema, chafing, ciliary sulcus, intraocular lens, hydrophilic acrylic IOL

## Abstract

**Background/Objectives**: this study aims to assess the safety of ciliary sulcus-placed hydrophilic acrylic intraocular lenses (IOLs). **Methods:** In this retrospective cohort study, consecutive patients who underwent phacoemulsification with implantation of IOLs into the ciliary sulcus without suture fixation between 2014 and 2016 at the Meir Medical Center were included. Clinical outcomes were compared between one-piece (1P) hydrophilic acrylic IOLs (Seelens AF, Hanita Lenses, Kibbutz Hanita, Israel) and three-piece hydrophobic acrylic IOLs with PMMA haptics (3P) (MA60AC, Alcon Laboratories, USA). **Results:** Thirty-eight eyes met the inclusion criteria and had ciliary sulcus IOLs implanted, twenty-three eyes with 1P hydrophilic (60.52%) and fifteen (39.47%) with 3P hydrophobic IOLs. Mean follow-up was 47.36 ± 7.25 months for the 1P group and 46.54 ± 9.82 months for the 3P group (*p* = 0.87). The mean peak IOP was higher in the 3P group (*p* = 0.038). No differences in the incidence of anterior uveitis or cystoid macula edema (CME) were detected between the groups (*p* > 0.05). None of the patients in our study developed uveitis, bleeding episodes, or required treatment for increased intraocular pressure, and no patient was diagnosed with uveitis-glaucoma-hyphema (UGH) syndrome. Post-operative corrected distance visual acuity (CDVA) was similar between the groups (*p* = 0.66). **Conclusions:** Hydrophilic IOLs can be safely placed in the ciliary sulcus and are non-inferior to the implantation of three-piece hydrophobic IOLs in the sulcus. In our cohort, with an average follow-up of approximately four years, no UGH was diagnosed, and none of the lenses were explanted.

## 1. Introduction

A total of 30 million cataract surgeries are performed annually worldwide [1]. While cataract surgeries are generally considered safe and the anatomical relations of the eye tissues could be adequately preserved, in some occasions, the capsular bag support is absent, and reconsideration needs to be performed regarding the appropriate intraocular lens (IOL) model to maintain the functionality of the optical system in the eye [2,3,4].

Chafing of the uveal tissue by IOLs may cause inflammation, bleeding, increased intraocular pressure, and iris atrophy with pigment dispersion, which may further cause short- and long-term complications such as cystoid macular edema (CME), synechiae, glaucoma, and even blindness [5,6,7]. The phenomena mentioned above were initially described in relation to the implantation of rigid polymethyl methacrylate (PMMA) IOLs into the anterior chamber [8,9]. The syndrome, initially described by Ellingson, had appeared within months after surgeries, and was estimated to occur in 2.2% of the cases annually. To mitigate tissue damage, the edges of the PMMA lenses were rounded via tumble polishing. The incidence of uveitis-glaucoma-hyphema syndrome has decreased significantly due to the shift away from anterior chamber IOLs, and it is currently believed to have a prevalence of around 0.4–1.2% yearly [10].

With this paradigm shift, it transpired that these tissue damages may also happen with posterior chamber IOLs and other intraocular devices that may cause mechanical chafing, such as sulcus-supported IOLs, sutures and other fixation devices, Soemmerring rings, filtration devices, and even in-the-bag IOLs, when the whole complex is unstable [11,12,13,14,15].

As cataract surgeries have evolved and foldable acrylic IOLs were introduced to the market, many polymeric composites have been developed, and many IOL designs are available nowadays. Foldable acrylic IOLs are divided into two main groups: (1) hydrophilic acrylic IOLs, which are made with a higher content of water, are generally softer, and are usually made as a single-piece IOL (1P) (these are less commonly used in large parts of the world, i.e., the USA and Canada), and (2) hydrophobic acrylic IOLs, which can be further divided into 1P IOLs, which are made as a single molding of the polymer that is curved into a certain shape, and three-piece IOLs (3P), which contain two rigid haptics and a foldable acrylic optic. The most common complication of capsular-fixated IOLs is posterior capsule opacification (PCO), which is largely affected by lens design. Sharp, square-edged IOL designs significantly reduced the PCO rate; however, the direct contact of the sharp edge with the delicate uveal tissue was associated with a sharp increase in uveitis-hyphema-glaucoma (UGH) syndrome [16,17,18,19].

These evolvements, together with a significant prevalence of iris chafing and derived complications (of which UGH syndrome is a known representative example), led several researchers to conclude that single-piece IOLs, placed in the ciliary sulcus, constitute a significant risk factor for these complications [20,21]. This conclusion is based on studies conducted mostly with hydrophobic IOLs and did not take into account hydrophilic IOLs (as they are less commonly used in vast parts of the world). Therefore, conclusions from these studies could not be deduced for the latter.

This issue remains incompletely resolved, as long-term evidence-based data addressing it are scarce. A recent retrospective study by Torrefranca et al. compared 6-month results of 1P sulcus IOLs, both hydrophilic and hydrophobic, with 3P sulcus IOLs and showed no significant differences in various outcome measures between the groups [22]. We used different types of lens materials, including hydrophilic and hydrophobic lenses, and clinically observed along the years that hydrophilic IOLs placed in the ciliary sulcus are associated with significantly less iris chaffing than hydrophobic IOLs, even when the lens has a single-piece, square-edge design.

This study aimed to assess the short- and long-term safety of ciliary sulcus-placed hydrophilic acrylic IOLs compared to hydrophobic 3-piece IOL lenses, which are considered suitable for sulcus fixation.

## 2. Materials and Methods

In this retrospective cohort study, we reviewed files of all consecutive cataract extraction patients who underwent cataract surgery at the Meir Medical center between January 2014 and December 2016. We included patients in whom the IOLs were implanted into the ciliary sulcus with no additional fixation. Clinical outcomes were compared between 1P hydrophilic acrylic IOLs (Seelens AF, Hanita Lenses, Kibbutz Hanita, Israel) and 3P hydrophobic acrylic IOLs with PMMA haptics (3P) (MA60AC, Alcon Laboratories, Fort Worth, TX, USA).

Ethics: this study was approved by the Institutional Review Board of Meir Medical and adhered to the tenets of the Declaration of Helsinki.

**Figure 1:**
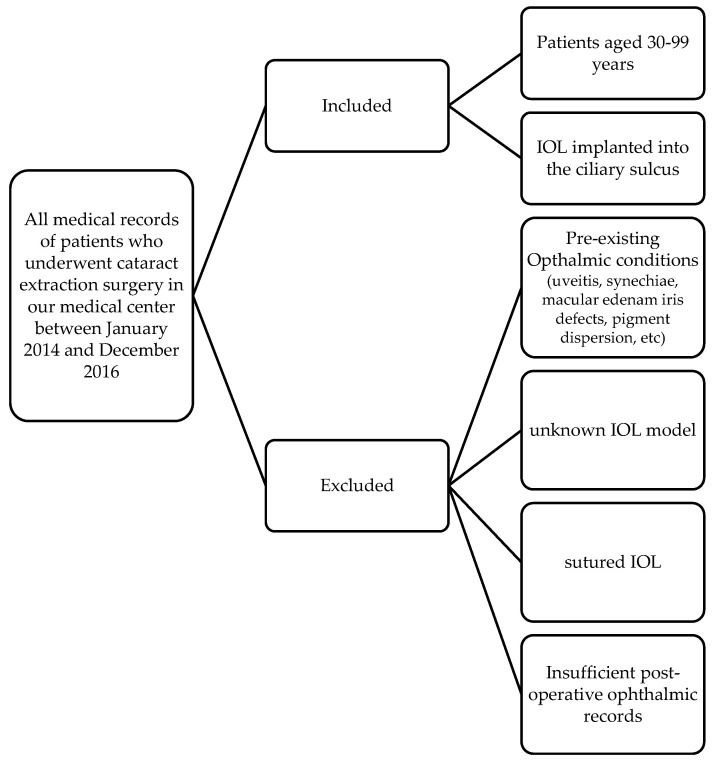
Inclusion and exclusion criteria.

Inclusion and exclusion criteria: We reviewed all medical records of patients who underwent cataract extraction surgery in our medical center between January 2014 and December 2016. Patients aged 30 to 99, in whom the IOL was implanted into the ciliary sulcus during surgery, were included. Patients who did have a previously reported ophthalmic condition that may resemble iris chafing and its complications (uveitis, synechia, macular edema, iris defects, pigment dispersion, etc.), patients with no record in their file of the IOL model being implanted, patients with suture fixation of the IOLs, and patients with insufficient post-operative ophthalmic medical records were excluded. Figure 1 explains the inclusion and exclusion criteria.

Surgical technique: Cataract extraction was performed using small incisions and phacoemulsification systems (Infinity by Alcon Laboratories, Fort Worth, TX, USA). With the recognition of the inability to implant IOL into the capsule, and with the reassurance of a sufficient anterior capsular support, after taking all necessary measures (i.e., lens material removal, anterior vitrectomy, ciliary sulcus reconstruction with viscoelastic materials, etc.), a foldable acrylic IOL was implanted in the ciliary sulcus. The IOL model to be implanted was at the discretion of the surgeon.

Data gathering: Data collected in this study included demographic characteristics, medical history, preoperative evaluation findings, surgical reports, and post-operative evaluation in ophthalmic clinics and tertiary ophthalmic centers. Data regarding the surgical course, intra-operative complications, and patient cooperation during the surgery were collected from the surgical report and were treated as numeric (for cooperation, a conversion to a binary parameter was performed). We retrieved data about the patients from all clinic visits and checked for events related to UGH and other common complications such as whiteout events on anamnesis, hyphema, anterior chamber cells, Trans-illumination defects (TIDs), and CME. The data presented in the post-operative period comprise all the follow-up visits of the patients included in the study. While the patients that were included in the study underwent cataract extraction and intraocular implantation surgery between 2014 and 2016, data collection was conducted between the years 2021 and 2022, during which all relevant data from the time of surgery until the medical chart review were included. Numeric data (intraocular pressure (IOP), visual acuity (VA), etc.), were averaged during the whole follow-up period, and the prevalence of adverse events was summed for the whole period.

Statistical analysis: Statistical analyses were performed using the JMP program (version 17.0, SAS, Cary, NC, USA). For categorical variables, the χ^2^ test was used. Fisher’s exact test was used for categorial variables with a cell frequency below 5. For continuous variables, we employed the independent *t*-test, which is considered robust for comparing groups of our sample sizes. *p*-values less than 0.05 on a two-sided test were considered statistically significant. Unless otherwise specified, data are presented as mean ± standard deviation (SD). Visual acuity values were converted to LogMAR values for statistical analysis. Patients with vision of light perception or worse were excluded from visual acuity analysis.

## 3. Results

Thirty-eight eyes met the inclusion criteria and were included in our analysis, twenty-three with 1P (60.52%) and fifteen (39.47%) with 3P. The mean follow-up was 47.36 ± 7.25 months for the 1P group and 46.54 ± 9.82 months for the A3P group (*p* = 0.87). No statistically significant differences were found concerning patients’ age, gender, and CDVA (*p* > 0.05 for all). Demographic and basic data are presented in Table 1.

The IOL was implanted into the ciliary sulcus due to a posterior capsule tear in 21 eyes (91%) of the 1P group and in 15 eyes (100%) of the 3P group (*p* = 0.26). In one case in the 1P group, an Argentinian flag tear led to the placement of the IOL in the sulcus, and in one case, the reason was an impression of zonular weakness (4.35% each).

The duration of the surgery was 46.52 ± 18.77 min in the 1P group compared to 69.41 ± 30.45 in the 3P group (*p* = 0.015). Anterior vitrectomy was performed in 80% of the surgeries of the 3P group and 65% of the 1P group (*p* = 0.33). Except for one patient in the 1P group, who had a combined surgery with trabeculectomy, all cases were a stand-alone cataract extraction.

Data on intra-operative characteristics is detailed in Table 2.

A significant difference in maximal IOP was found between the groups, as higher maximal pressure was documented in the 3P group (24.07 ± 10.57 versus 18.48 ± 5.92, *p* = 0.0384). Nevertheless, no significant difference was demonstrated in the mean cup-to-disk ratio, the need for the addition of hypotensive treatment, and the documentation of non-post-operative (defined as the first month following cataract extraction procedure) anterior uveitis, hyphema, TIDs, or CME (*p* > 0.05 for all). Table 2 elaborates all post-operative data.

## 4. Discussion

The implantation of a posterior chamber IOL in the sulcus can be associated with UGH syndrome and related pathologies and can even lead to blindness.

In our study, no patient in either group developed significant complications after the implantation of an IOL to the ciliary sulcus. The surgery duration was shorter in the 1P group, suggesting that the implantation of the 1P hydrophilic IOL to the ciliary sulcus is easier than the implantation of a 3P IOL (this could be derived from the need to enlarge the main surgical wound for the 3P cartridge to fit in, to maneuver the IOL and its more rigid haptics into the sulcus, and to the suturing of the enlarged wound which may be needed in some cases). The post-operative results were similar between the groups with low complication rates and similar visual acuity improvement. Due to the retrospective nature of this chart review, with no pre-set follow-up protocol, we could not accurately document the duration of any of the post-operative findings (elevated IOP, persistent inflammation, CME, or others). We assumed that prolonged events would be reflected in appropriate diagnoses and secondary manifestations (for example, increased cupping of the optic nerve, receiving a diagnosis of glaucoma, etc.).

The existing data and clinical observations (based majorly on data arising from leading countries in North America) led to the prevailing perception that the implantation of a single-piece IOL in the ciliary sulcus is hazardous, is highly correlated with the development of UGH syndrome, and should be generally avoided. In the UGH article within the website “EyeWiki”, a digital encyclopedia of the American Academy of Ophthalmology, it is stated that “Single-piece acrylic IOLs placed within the sulcus tend to have a high UGH complication rate”. The recommendation of the authors is as follows: “A 1 piece lens should not be placed in the sulcus. If there is enough support a 3-piece lens should be placed within the sulcus” [23]. Chang et al. stated that they would recommend avoiding SPA implantation to the ciliary sulcus, including single-piece hydrophilic acrylic IOLs declared by the manufacturer as appropriate for sulcus placement, due to their planar, square-edged design. That is, their study included only one hydrophilic acrylic IOL [24].

In the vast majority of the industrialized countries worldwide, the most common IOLs used in standard cataract surgeries are different designs of square-edge 1P hydrophobic acrylic IOLs, which were proven to give durable, long-lasting, and good optical results with good biocompatibility. The recognition of the fact that a square-edge design of the optic is more appropriate to lower rates of late PCO, which is one of the most common complications of modern cataract surgeries nowadays; many manufacturers shifted to the aforementioned design [16,25,26,27]. After it was proven in a series of studies that 1P hydrophobic IOLs placed in the sulcus may constitute a significant risk factor for iris chafing, resulting in UGH syndrome [20,28,29,30], which might have rueful results, leading to vision loss and even blindness, most surgeons shifted to implant 3P IOLs in the sulcus when the posterior capsular support could not be relied on. In the case of a posterior capsule (PC) tear, as well as the presence of good zonular support and a full continuous curvilinear anterior capsule rhexis, the common practice nowadays is to implant a 3P hydrophobic acrylic IOL to the ciliary sulcus, with or without an optic capture [31,32]. The advantage of using a single-piece hydrophilic lens over a 1P hydrophobic lens would be better shown if we compared the two types of lenses in a single randomized trial. However, since the high rate of UGH in eyes with sulcus-fixated hydrophobic IOLs is so well documented, it would be not ethical to include such a group in our study, and we had to compare our clinical results to historical data. In a study on IOL explantation in Chinese patients by Chan et al., it was found that “Sulcus implantation of a single-piece acrylic (SPA) IOL resulted in UGH syndrome in all cases, while sulcus-fixated 3-piece lenses had such complication in only 7.1% of cases” [33].

Since 3-piece acrylic IOLs implantation is proven to be superior to a 1P hydrophobic sulcus fixated lens, a 1P IOL that would provide clinical results that are at least comparable to 3P lenses could be considered superior to a 1P hydrophobic IOL placed in the ciliary sulcus.

It is important to note that 3P IOLs also have different models, designs, and composing materials, which contribute to different safety profiles among them. Comparison between different 3P IOL models is beyond the scope of this article; therefore, we included the only 3P IOL model that was available in our surgical center, and it is considered the benchmark sulcus implantation in the case of lacking capsular support in our region.

It is known that the IOL design has a great impact on its function, both mechanically and optically. While a lot was studied about different designs of IOLs in different contexts (i.e., optics’ edge in relation to the formation of posterior capsular opacifications and the optical effect it produces, the optic and haptic structures and their relation to the capsulorhexis in the context of dysphotopsias, and more), only scarce data could be found about the correlation between IOLs’ design, the development of UGH, and related complications, and the common belief that implanting a single piece foldable IOL to the ciliary sulcus is a poor decision was rooted [34].

In our country, unlike some of the other markets, hydrophilic IOLs are common and are widely used in both routine and complicated cases. This allowed us to gain a wide experience with the implantation of different hydrophilic acrylic models and designs of IOLs, which in turn led to the clinical observation that UGH syndrome is very rare with the implantation of those.

Data supporting the safety of the presence of the hydrophilic acrylic polymer itself in the ciliary sulcus was established with the assessment of hydrophilic acrylic piggyback IOL implantation [35,36], as well as of hydrophilic acrylic posterior chamber phakic IOLs [37].

Hanita SeeLens AF is a relatively soft and flexible double square edge aspheric hydrophilic IOL, with relatively thin double C loop haptics (3.1 mm) and a 5-degree posterior angulation between the haptics and the optic, which was originally designed to be placed in the capsular bag and the ciliary sulcus (see company commercial information). It is widely used in Europe, Africa, Latin America, Asia, and Oceania.

Bar-Sela and Fleissig published intermediate-term results of a series of 13 patients with PC tears who were implanted with SeeLens AF in the ciliary sulcus [38]. In their series, no significant complication occurred, and CDVA was improved in all cases. The hypothesis in their study was that the design of the IOL (i.e., hydrophilic acrylic material, thin and angulated haptic, and large overall diameter) is what makes these IOLs suitable for implantation in the sulcus. They concluded that this IOL model gave good final CDVA with low rates of complications.

In our department, a high volume of cataract surgeries is performed each year by different surgeons of different skill levels. Over the years, we have gained experience in implanting a hydrophilic acrylic IOL into the sulcus and got the clinical impression that this is an effective and safe solution.

Our study demonstrates that the implantation of a 1P hydrophilic IOL to the ciliary sulcus is non-inferior to the implantation of 3P hydrophobic IOLs in cases of PC tear, and it may even have advantages compared to implanting a 3P IOL in the same location, giving a shorter surgery duration.

For methodological and simplicity reasons, we reported our data with a single model of a hydrophilic acrylic IOL, but our experience showed similar results with other single-piece hydrophilic acrylic IOLs (such as Biflex 677AD, Medicontour Medical Engineering Ltd., Zsambek, Hungary, and Rayone, Rayner Ltd., Worthing, West Sussex, UK). Based on our empirical observations, not only the IOL design but also the inherent hydrophilic properties of the polymer material appear to play a significant role in the suitability of an IOL for placement within the sulcus. Thorough evaluations must be conducted on these IOL models, along with similar variants, to establish evidence-based conclusions about their safety profile.

It is important to note that UGH is a fairly rare complication as a whole, and it may present only in parts and may also develop at a late time after the surgery. Yet, in our study, with a long-term follow-up of several years, even milder adverse events or complications occurred in a minority of the patients in both groups, with similar rates between the two groups. This emphasizes the fact that the sulcus, as a target for IOL implantation, gives safe and effective long-term results when an appropriate IOL model is used.

Our study has some limitations. First, it is retrospective, which makes it exposed to some biases. Second, our study groups are relatively small. This stems from the fact that we strived to achieve a long-term follow-up cohort with sufficient medical documentation. Since a PC tear is not very common and considering that implanting a 1P IOL to the sulcus is not a common practice, we believe that this is a good representing group for a primary validation study, which could pave the way for further investigation in this field, which is warranted. To the best of our knowledge, this is the biggest reported cohort with such long-term data. Last, the use of Seelens AF might not correctly represent other models of IOL, and further studies should be conducted to confirm our clinical observations.

## 5. Conclusions

Our study demonstrates the long-term safety and efficacy of a sulcus-fixated hydrophilic acrylic one-piece IOL, “Seelens AF”, with no cases of UGH syndrome due to uveal chafing observed. Despite limitations, these findings support the use of hydrophilic acrylic IOLs as a viable option for sulcus implantation.

This study challenges the prevailing axiom, suggesting that IOL material properties and edge design, rather than single-piece structure alone, are critical determinants of sulcus implantation safety. Our findings indicate that carefully selected IOL models, including certain hydrophilic single-piece designs, can be safely and effectively implanted in the ciliary sulcus, potentially expanding surgical options in cases lacking adequate capsular support.

## Figures and Tables

**Table 1:** Demographic data, pre-operative, and intra-operative characteristics.

	3P	1P	*p* Value
Number of eyes	15	23	
Gender—female (%)	7 (46%)	18 (78%)	0.047
Age	74.64 ± 8.409	77.57 ± 7.14	0.150
Laterality—Right eye	8 (53.33%)	10 (43.47%)	0.550
Pre-operative measurements			
Cataract staging			
Nuclear sclerosis	1.933 ± 1.163	2.34 ± 0.88	0.227
Posterior sub capsular	0.733 ± 1.223	1 ± 1.314	0.530
Cortical cataract	0.533 ± 0.915	0.87 ± 0.92	0.227
Spherical equivalent	0.167 ± 3.698	−0.833 ± 2.758	0.460
Axial Length	23.409 ± 1.030	23.003 ± 0.945	0.330
Anterior chamber depth	3.604 ± 0.725	3.046 ± 0.437	0.019
CDVA (logMAR)	0.85 ± 0.39	0.86 ± 0.48	0.966
Maximal medicated pupil diameter	7.0 ± 1.24	6.9 ± 1.48	0.579
PXF	2 (13.3%)	4 (17%)	0.740
Intra-operative data			
Surgery duration	69.41 ± 30.45	46.52 ± 18.77	0.015
IFIS	0 (0%)	1(4.3%)	0.495
Anterior vitrectomy	12 (80%)	15 (65%)	0.332
Phacodonesis	0	1 (4.3%)	0.498

CDVA—corrected distance visual acuity, IFIS—intra-operative floppy iris syndrome, PXF—pseudoexfoliation syndrome.

**Table 2:** Post-operative data and complications.

	3P	1P	*p* Value
Data during follow up			
Maximal IOP	24.07 ± 10.57	18.48 ± 5.92	0.038
Hyphema	1 (6.67%)	0 (0%)	0.216
Anterior chamber cells	4 (26.6%)	3 (13%)	0.296
TIDs	1 (6.67%)	3 (13%)	0.536
Whiteout events	0 (0%)	0 (0%)	1
CME	1 (6.67%)	2 (8.7%)	0.823
Vitrectomy for dropped lens particles evacuation	2 (13.3%)	1 (4.3%)	0.325
Vitrectomy for retinal detachment repair	2 (13.3%)	0	0.075
UGH diagnosis	0 (0%)	0 (0%)	1
Data on last visit			
Synechiae	2 (13.3%)	3 (13%)	0.979
CDVA on the last visit (logMAR)	0.67 ± 0.63	0.58 ± 0.55	0.66
CDVA change (compared to pre-operative)	+0.28 ± 0.84	+0.29 ± 0.833	0.96
IOL tilt	0	2 (8.6%)	0.247
IOL decentration	1 (6.67%)	1 (4.3%)	0.757
Cup to disk ratio	0.267 ± 0.322	0.283 ± 0.316	0.851
Anti-glaucoma treatment addition	3 (20%)	3 (13%)	0.571

IOP—intraocular pressure, TIDs—trans-illumination defects, CME—cystoid macular edema, CDVA —corrected distance visual acuity, IOL—intraocular lens, UGH—uveitis-glaucoma-hyphema.

## Data Availability

The data presented in this study are available on request from the corresponding author.

## Abbreviations

The following abbreviations are used in this manuscript:

IOLs	Intraocular lenses
1P	One-piece
3P	Three-piece hydrophobic-acrylic IOL with PMMA haptics
CME	Cystoid-macula-edema
UGH	Uveitis-glaucoma-hyphema
CDVA	Corrected distance visual acuity
PMMA	Polymethyl methacrylate
PCO	Posterior capsule opacification
TIDs	Trans-illumination defects
IOP	Intraocular pressure
VA	Visual acuity
SD	Standard deviation
IFIS	Intra-operative floppy iris syndrome
PXF	Pseudoexfoliation syndrome
PC	Posterior capsule
SPA	single-piece acrylic
